# Effects of Electromyography Bridge on Upper Limb Motor Functions in Stroke Participants: An Exploratory Randomized Controlled Trial

**DOI:** 10.3390/brainsci12070870

**Published:** 2022-06-30

**Authors:** Qin Zhao, Gongwei Jia, Lang Jia, Yule Wang, Wei Jiang, Yali Feng, Hang Jiang, Lehua Yu, Jing Yu, Botao Tan

**Affiliations:** 1 Department of Rehabilitation Medicine, The Second Affiliated Hospital of Chongqing Medical University, Chongqing 400010, China; 2020140156@stu.cqmu.edu.cn (Q.Z.); 302145@cqmu.edu.cn (G.J.); 302116@hospital.cqmu.edu.cn (L.J.); wangyule@hospital.cqmu.edu.cn (Y.W.); 301194@hospital.cqmu.edu.cn (W.J.); 304513@hospital.cqmu.edu.cn (Y.F.); 304772@hospital.cqmu.edu.cn (H.J.); 300895@cqmu.edu.cn (L.Y.); 2 Department of Rehabilitation Medicine, The First Affiliated Hospital of Jinan University, Guangzhou 510630, China

**Keywords:** stroke, hemiplegia, electromyographic bridge, upper limb rehabilitation, randomized controlled trial

## Abstract

The electromyography bridge (EMGB) plays an important role in promoting the recovery of wrist joint function in stroke patients. We investigated the effects of the EMGB on promoting the recovery of upper limb function in hemiplegia. Twenty-four stroke patients with wrist dorsal extension dysfunction were recruited. Participants were randomized to undergo EMGB treatment or neuromuscular electrical stimulation (NMES). Treatments to wrist extensors were conducted for 25 min, twice a day, 5 days per week, for 1 month. Outcome measures: active range of motion (AROM) of wrist dorsal extension; Fugl-Meyer assessment for upper extremity (FMA-UE); Barthel index (BI); and muscle strength of wrist extensors. After interventions, patients in the NMES group had significantly greater improvement in the AROM of wrist dorsal extension at the 4th week and 1st month follow-up (*p* < 0.05). However, patients in the EMGB group had a statistically significant increase in AROM only at the follow-up assessment. No significant differences were observed in the AROM between the EMGB group and the NMES group (*p* > 0.05). For secondary outcomes in the EMGB group, compared to baseline measurements, FMA-UE, BI, extensor carpi radialis and extensor carpi ulnaris muscle strength were significantly different as early as the 4th week (*p* < 0.05). The muscle strength of the extensor digitorum communis muscle showed significant differences at the follow-up (*p* < 0.05). There were no statistically significant differences between patients in the two groups in any of the parameters evaluated (*p* > 0.05). The combination of EMGB or NMES with conventional treatment had similar effects on the improvement of the hemiplegic upper limb as assessed by wrist dorsal extension, FMA-UE, and activities of daily living. The improvement in both groups was maintained until 1 month after the intervention.

## 1. Introduction

Stroke is the leading cause of adult disability worldwide [1]. More than 50% of stroke survivors exhibit permanent neurological impairments, with motor impairment being the most frequent. Even after standard neurological rehabilitation, approximately 80% of these patients suffer from hand dysfunction [2]. The increased muscle tension of the wrist dorsal extension in stroke patients severely affects the active range of motion (AROM) and the wrist dorsal extension function, and wrist joint dysfunction directly affects the motor control function of the upper limb [3]. The improvement of wrist control improves the quality of life for stroke survivors, reduces comorbidities associated with a loss of independence, and reduces the costs associated with the healthcare system [4,5,6]. Neuromuscular electrical stimulation (NMES) is one of the most common strategies for improving limb function in the clinical setting. Studies have shown that NMES can improve muscle strength, reduce spasticity, increase joint range of motion by promoting active movement, reorganize the damaged cortico-cerebral circuit, and improve movement control [7,8,9,10]. However, during the NMES process, the hemiplegic limb is passively moved, and bilateral limbs exhibit no movement to work together, which greatly reduces the effect of the patient’s rehabilitation training. Therefore, the combined modulation of bilateral movement and electrical stimulation potentially play an important role in enhancing patient noncoordinated movement [11,12].

Recently, the State Key Laboratory of Bioelectronics at Southeast University developed a new type of self-controlled NMES system: the electromyography bridge (EMGB) [13,14]. In this instrument, the surface electromyography (sEMG) signals of the nonhemiplegic muscles are converted to control the duration and frequency of the relevant stimulation pulses applied to the muscles of the hemiplegic side. Therefore, the activation state of the control muscles can better simulate the coupling of bimanual exercises and movement responses. EMGB combined with NMES is effective in the short-term for improving upper limb injury in patients with stroke [15]. In a study of eight healthy subjects, EMGB accurately reproduced voluntary muscle forces and was more resistant to fatigue than NMES [13]. Some previous reports have shown that EMGB plays a certain role in promoting the recovery of wrist joint function in stroke patients [16,17], but the results remain controversial. Stroke patients were treated with EMGB for 4 weeks within 6 months of onset. The voluntary surface electromyographic ratio of wrist and finger extensors, Brunnstrom stages for the hand, and FMA-UE were significantly improved compared with the NMES group [16]. However, other articles showed that when thirty-one stroke patients received three weeks of EMGB or NMES treatments, the two treatment modalities showed no significant difference in FMA-UE, self-care FIM, grip strength, or on the modified Ashworth scale [18]. The number of studies of EMGB for stroke patients is limited. The effect of EMGB on wrist function in stroke patients is yet to be elucidated.

Between 1 week and 6 months post-stroke (subacute period of stroke) is a critical time for neural plasticity; most behavioral recovery and rapid changes occur in the first weeks and months post-stroke for the majority of people [19]. Therefore, this study aimed to compare the effects of EMGB and NMES on the recovery of upper limb motor and functional performance in subacute rehabilitation.

## 2. Materials and Methods

### 2.1. Study Design

The study was designed as a 2:1, double-blinded, randomized controlled trial. All subjects received 40 treatments twice a day, 5 days per week, for 4 weeks. The assessments were made at baseline, at the 4th week during treatment, and at the 1st month after discharge by a blinded therapist.

### 2.2. Participants and Setting

Stroke patients who participated in this work suffered from unilateral upper limb hemiparesis, and they could not dorsally extend their wrists. They were hospitalized at the Rehabilitation Medicine and Physical Therapy Department at the Second Affiliated Hospital of Chongqing Medical University between May 2020 and January 2021. The protocol of this study was registered with the Chinese Clinical Trial Registry (registration number: ChiCTR2100051957). The inclusion criteria were as follows: (1) stroke diagnosis that was confirmed by evidence on computed tomography (CT) or magnetic resonance imaging (MRI) scans 3 days to 12 months before the study; (2) a patient of any sex who was aged between 18 and 75 years; (3) poor upper limb function (AROM of the wrist extension was 0°), although the contralateral upper limb functioned well; (4) no progressive stroke and stable vital signs; and (5) the ability to understand and agree to the trial procedures and to sign an informed consent form in accordance with national legislation. Patients with any of the following criteria were excluded: (1) severe cognitive disorders [20] (Mini-Mental State Examination score ≤16); (2) severe depression [21] (Hamilton Rating Scale for Depression (HAMD) ≥24); (3) a modified Ashworth scale (MAS) score of two or more points for spasticity in carpal extension; (4) carpal contracture; (5) New York Heart Association (NYHA) cardiac function was classified as Level 4; (6) alanine aminotransferase (ALT) and aspartate aminotransferase (AST) were double the upper limit of normal; (7) severe heart, liver, kidney or lung diseases, or cancer; (8) cardiac pacemakers and various implantable electronic devices; (9) pregnant or lactating women; (10) muscles do not respond to functional electrical stimulation (FES); (11) concurrent participation in another similar clinical study during the 3 months before enrollment; and (12) other reasons deemed by the investigators to render the subject unsuitable for this trial.

### 2.3. Procedure

**Figure 1:**
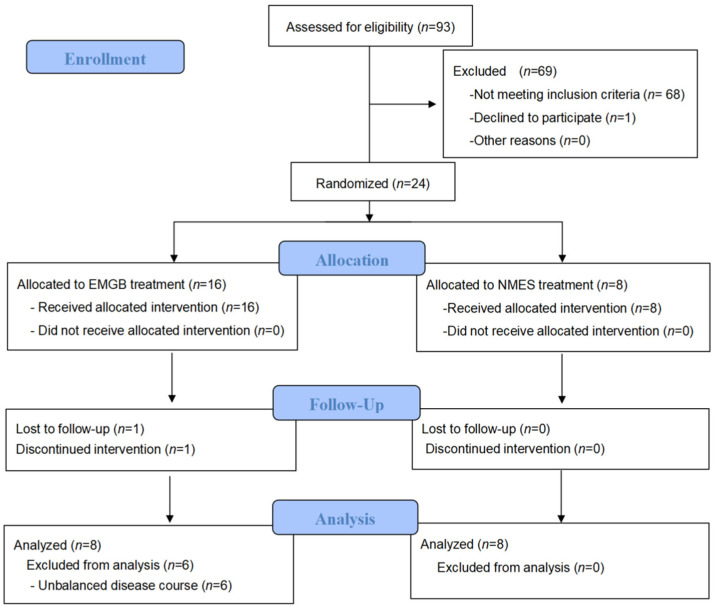
The CONSORT patient flowchart.

Out of the 93 patients screened, 24 patients agreed to join the group and were randomized into either the EMGB group (*n* = 16) or the NMES group (*n* = 8). The plan accepted by each patient was determined by random allocation. The members of the different groups were recorded in order in a table. The patients’ group assignments documented in the table were covered by an opaque coating. Only after scratching off the coating were the patients informed of which group they were assigned to. Two patients in the EMGB group who were discharged and failed to complete follow-up were excluded from the study. Patients in the two groups were well-matched in age, sex, stroke type, hemiplegia side, muscle tone, and HAMD score at baseline. However, the course of stroke onset was unbalanced at baseline (*p* < 0.05). After a detailed analysis of the patients’ information, we found that there were 6 patients in the EMGB group who had a relatively long course of stroke (>6 months). Therefore, we removed those patients for further analysis (Table 1). As a result, all remaining patients had similar courses of stroke (ranging from 1–6 months), and other characteristics were also comparable at baseline [22]. The complete data are shown in Supplementary Materials (Tables S1–S3). The Consolidated Standards of Reporting Trials (CONSORT) patient flowchart is shown in Figure 1.

#### 2.3.1. EMGB Group

We used the double-channel motor function reconstruction instrument based on the EMGB principle as in previous studies [23]. Similarly, patients were seated in front of a desk with their upper limbs attached to this system. Their elbows were flexed naturally, and their wrists were pronated, fully exposing both forearms. For sEMG detection, the sEMG sensors were placed on the wrist extensors of the nonhemiplegic limb to collect sEMG signals. The stimulation electrode was fixed to the optimal stimulation points for wrist extensors of the hemiplegic limb at a stimulating intensity below the threshold for uncomfortable sensation. A gelled reference electrode was placed over the olecranon. Patients were asked to perform simultaneous bilateral wrist extensions with a cycle comprising 5 s extension and 5 s relaxation periods under the guidance of a rhythmic sound generated by a recorder. The training consisted of two sessions of 10-min EMGB use with a 5-min interval between sessions. Sessions were conducted twice a day (once in the morning and once in the afternoon) for 4 weeks.

#### 2.3.2. NMES Group

The patients were in the same position as the patients undergoing EMGB treatment. The sEMG sensors were placed on the wrist extensors of the hemiplegic limb to collect sEMG signals. A dorsal wrist extension of the hemiplegic side was passively elicited by preprogrammed NMES with the same sound cues.

**Figure 2:**
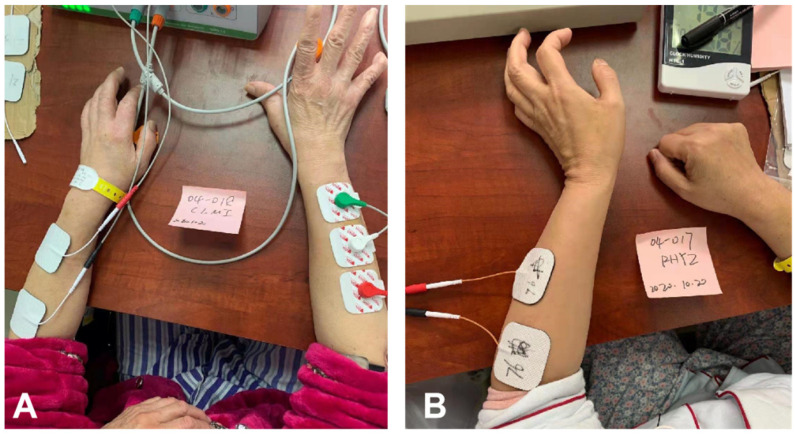
Electromyography bridge(EMGB) group and neuromuscular electrical stimulation(NMES) group. (**A**) Patients undergoing EMGB treatment; (**B**) Patients undergoing NMES treatment.

Licensed therapists with at least 3 years of clinical experience performing manipulative therapies provided all treatments. None of the patients in either group experienced serious adverse effects. In addition to EMGB or NMES treatments, patients were offered conventional therapy (such as manual treatment and acupuncture). Figure 2A,B show the setup for the training tasks completed by patients in the 2 groups.

### 2.4. Outcome Measurement

Evaluations were performed at baseline, at the 4th week and at the follow-up after the 1st month of the trial by a therapist who had no information about the treatment groups. The primary outcome was an assessment of joint mobility using AROM of wrist dorsal extension. All patients had an AROM of 0° at enrollment. The secondary outcomes were as follows: (1) the Fugl-Meyer Assessment for Upper Extremity (FMA-UE) was used to evaluate motor recovery and motor function of upper limbs; (2) the manual muscle test (MMT) was used to evaluate the muscle strength of the extensor wrist muscles to evaluate muscle function around the wrist; and (3) the Barthel index (BI) was used to assess ADL performance.

### 2.5. Statistical Analysis

Statistical analyses were performed using SPSS 26.0 (SPSS Inc., Chicago, IL, USA). Data are expressed as the mean ± SD (standard deviation). Before performing the comparisons, we tested the data to determine whether they were normally distributed, and the variances were equal. The Shapiro–Wilk test was used to evaluate the data of the measurable parameters for a normal distribution in each group. To compare the baseline characteristics between the 2 groups, Fisher’s exact tests and independent sample *t* tests were used to analyze variables. Repeated-measures ANOVA was used to compare the AROM of wrist dorsal extension, FMA-UE, MMT, and BI at baseline, at the 4th week, and at the follow-up after the 1st month with 95% confidence intervals. *p* values < 0.05 were considered statistically significant.

## 3. Results

### 3.1. Patient Characteristics

Table 1 shows the baseline characteristics of the studied population. There were no significant differences between groups in most of the demographic data and baseline variables related to stroke and neurological status at the time of admission. The patients in the two groups were well-matched in age, sex, stroke type, hemiplegia side, and HAMD score.

### 3.2. Primary Outcomes

As shown by the mean changes from baseline (Table 2), AROM of wrist dorsal extension improvements were observed during the trial in both EMGB and NMES groups. AROM improvements in the NMES group were significantly different from baseline at the 4th week and at the follow-up after the 1st month. (The mean change from baseline was equal to 6.92° and 7.70°, *p =* 0.009 and 0.029, respectively.) The significant difference in EMGB was at follow-up after the 1st month. (The mean change from baseline was equal to 7.85°, *p* = 0.026.) Notably, improvement was observed in 8 patients (3/8, 37.50% in the NMES group and 5/8, 62.50% in the EMGB group) at the 4th week and in 10 patients (5/8, 62.50% in both the NMES and EMGB groups) at follow-up. At the follow-up after the 1st month, the AROM increase in the EMGB was slightly higher than that of the NMES (the mean difference between groups was equal to 0.15°, *p* = 0.97), but there was no significant difference between the EMGB and NMES groups.

### 3.3. Secondary Outcomes

Patients in both groups acquired functional recovery to some extent, as assessed by the FMA-UE, muscle strength of wrist dorsiflexion, and Barthel index.

**Figure 3:**
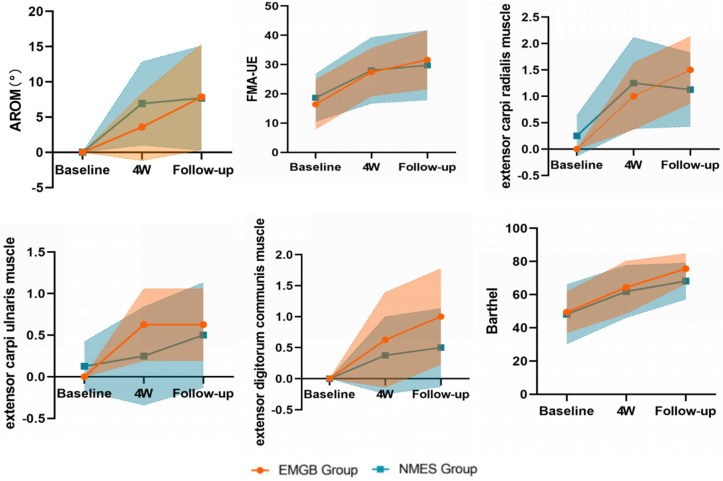
AROM: active range of motion; FMA-UE: Fugl-Meyer Assessment for Upper Extremity. The outcomes changed (mean and 95% CI) across the 3 time points between the EMGB and NMES groups. GraphPad Prism 8 was used to calculate the estimated marginal means: estimated marginal means (y-axis) for the EMGB group (orange); and NMES group (blue) across time points (x-axis).

Briefly, FMA-UE increased after intervention in both groups compared with baseline. Differences in changes from baseline were significant at the 4th week (*p* = 0.001 and 0.004, respectively) and follow-up after the 1st month (*p* = 0.001 and 0.007, respectively). No differences were seen at any time point between groups (Table 2, Figure 3).

The muscle strengths of three major wrist dorsiflexion (extensor carpi radialis muscle, extensor carpi ulnaris muscle, extensor digitorum communis muscle) are summarized in Table 2 and Figure 3. Similarly, the strength of the wrist extensor muscles improved in both groups at the 4th week (*p* = 0.009 for carpi radialis, and *p* = 0.023 for carpi ulnaris) and lasted at follow-up (*p* = 0.005 for digitorum communis) (Table 2, Figure 3). No significant differences were seen between the two groups at any time point.

Patients in both groups had improved activities of daily living as early as the 4th week (*p* = 0.006 and *p* = 0.01, respectively). Again, there were no significant differences between the groups at any time point (Table 2, Figure 3).

## 4. Discussion

In this study, we found that EMGB significantly improved the upper limb motor function of patients with subacute stroke. At the 1st-month follow-up, the AROM of wrist dorsal extension and extensor digitorum communis muscle of patients were significantly increased. Moreover, the FMA-UE score, muscle strengths of the extensor carpi radialis muscle and extensor carpi ulnaris muscle, and BI of patients were also significantly improved after 4 weeks of treatment. The results demonstrate that the improvement of wrist and upper limb function led by EMGB can be maintained for at least 1 month.

The instrument used in this research was a two-channel motor function reconstruction instrument for hemiplegic limbs. The instrument combines electromyography and the mean absolute value/number of slope sign changes (MAV/NSS) and co-modulation algorithm (MNDC) to control actions in real time through EMGB technology. Based on the principle of bilateral training, the sEMG of the nonhemiplegic limb was used to detect and collect the real-time motion status data and then generate stimulation pulses by the MNDC algorithm on the corresponding muscles of the hemiplegic limb; this way, the movements of the hemiplegic limb could be guided by the nonparalyzed side [13,24]. Other potential mechanisms of this new instrument for hemiplegia were recently investigated: (1) repeated intentional movement of the nonhemiplegic limb could activate the primary motor cortex of the hemiplegic side, which is helpful for establishing new motor neurofeedback to realize motor relearning and to increase the excitability and recruitment effect of the target muscle contraction on the hemiplegic side [25,26]; (2) EMGB is conducive to promoting the remodeling of the central neural network and triggering the function of movement [27,28]; (3) at the same time, noninvasive stimulation of EMGB increased peripheral blood flow and muscle strength [29,30]. This occurrence is the mechanistic basis for the functional recovery of stroke patients.

Some previous studies demonstrated the effectiveness of EMGB [17,31]. ZHOU [16] administered EMGB treatment to patients for 4 weeks, and the Brunnstrom stages for the FMA-UE, motor status scale, and voluntary sEMG ratio of the wrist and finger extensors of patients’ hemiplegic side were improved. The results favor EMGB treatment for augmenting the recovery of volitional wrist motion in stroke patients. Shini et al. [32] investigated the effect of EMG-triggered NMES on functional recovery of the affected hand and related cortical activity in chronic stroke. After the intervention of 10 weeks of EMG-triggered NMES, the hemiplegic hand showed significant improvements in the box and block test (BBT), strength, accuracy index (AI), and on/off set time of muscle contraction. These results suggest that EMG-triggered NMES could improve exercise capacity, exercise accuracy, and effective muscle recruitment in patients with hemiplegia. However, other research articles have different results. By comparing the effectiveness of active and passive neuromuscular electrical stimulation on the upper limbs of hemiplegia, no significant difference was detected in wrist extensor spasticity and upper limb functional between the two stimulation applications [18]. Hemmen and Seelen’s [33] study showed that EMG-triggered stimulation did not increase upper limb function recovery relative to NMES in subacute stroke patients. The results of these studies are consistent with our findings. Compared with NMES group, the outcome indicators of the EMGB group showed positive trends toward improved outcomes, but the trends were not statistically significant. Possible explanations for the lack of differential effects between the groups was the small sample size and the fact that only subacute patients were studied. EMGB treatment might accelerate recovery during the subacute stage, but the NMES group might catch up and have similar outcomes 6 months after stroke [22]. In addition, strict inclusion criteria (AROM of wrist dorsal extension was 0° at baseline) limited the number of patients. A total of 24 patients agreed to join our clinical trial, but only 16 patients could be included to balance the patients’ course of the disease. Another possible explanation was that conventional therapy was highly effective for the treatment of upper limb dysfunction, and other additional effects on wrist function were too small.

Most patients with stroke have better motor function in the proximal limb than in the distal upper limb. This outcome mainly occurs because patients exert more muscle strength with their shoulders and elbows when required to conduct upper limb movements [34,35]. This motor compensation develops new compensatory muscle activation patterns that differ from those of the unimpaired muscles. However, the flexibility of the hand and wrist has a great impact on the daily life of stroke patients. In some studies related to the rehabilitation of the distal joints, temporary paralysis of the proximal joint muscles was used to reduce the competition between the proximal and distal ends to obtain more distal muscle training [36]. In this research, it was found that EMGB provides a way to improve distal limb AROM. Deanna [37] quantified the ROM required for eight upper-extremity ADLs in healthy participants and found the activities required a total wrist motion of 38° of flexion, 40° of extension, 38° of ulnar deviation, and 28° of radial deviation. Brumfield and Champoux [38] reported that 10° of flexion and 35° of extension were required to accomplish most ADLs. In our study, the AROM of the wrist extension in both groups increased from 0° to approximately 8° at follow-up. They are still not able to complete most functional movements. Future studies should include longer intervention periods to achieve functional recovery.

This study had limitations. Firstly, there were only two groups in the current study, and no control group received conventional rehabilitation treatment alone. This was because patients in the control group would have received 50 min per day less treatment than the other groups, which was a significant medical ethical problem. Secondly, there was a limited number of patients. All participants included in our study were enrolled from a single center, and there was a relatively small sample size. Thirdly, in terms of the selection of evaluation indicators, we selected more indicators of body structure and function, but the level of activity was less-evaluated. As the activity level indicator, the Jebsen hand function test was not analyzed because most patients could not complete it. In addition, the scales used in this paper are ordinal, which means the ability to detect meaningful change may be impaired [39]. Future studies with larger populations, a multicenter clinical trial, and strict, stratified randomization are needed.

## 5. Conclusions

The findings of the present study suggested that EMGB might be beneficial to upper limb function recovery for patients with subacute stroke. However, our data did not show that EMGB had better effects than traditional NMES treatment in improving hemiplegic wrist extension, FMA-UE, and ADL performance. Further comprehensive studies should include a larger sample size and a longer observation period of stroke patients using balanced enrollment levels.

## Figures and Tables

**Table 1:** Clinical characteristics of the patients in both groups (long course removed).

	EMGB Group (*n* = 8)	NMES Group (*n* = 8)	*p* Value
Age, y	52.75 ± 17.16	53.88 ± 10.70	0.877
Sex, *n* (%)			
Male	5 (62.5)	5 (62.5)	1
Female	3 (37.5)	3 (37.5)	
Stroke type, *n* (%)			
Infarction	4 (50.0)	2 (0.25)	0.608
Hemorrhage	4 (50.0)	6 (0.75)	
Hemiplegia side			
Left	6 (0.75)	5 (62.5)	1
Right	2 (0.25)	3 (37.5)	
Muscle tone	0.38 ± 0.52	0.50 ± 0.53	0.642
HAMD	4.75 ± 6.82	5.50 ± 6.72	0.838
Stroke onset, month	1.38 ± 1.06	1.63 ± 1.06	0.645

EMGB: electromyography bridge; NMES: neuromuscular electrical stimulation; HAMD: Hamilton Rating Scale for Depression; y: year. Values are presented as the number of patients (*n*) or mean ± standard deviation. Significance difference at *p* < 0.05.

**Table 2:** Trial results for the primary and secondary outcomes.

		Baseline		Mean Difference between Groups (95%), *p* Value		4w		Mean Difference between Groups (95%), *p* Value		Follow-up		Mean Difference between Groups (95%), *p* Value	
		EMGB	NMES			EMGB	NMES			EMGB	NMES		
	*N*	8	8			8	8			8	8		
Primary Outcome	AROM	0.00 ± 0.00	0.00 ± 0.00	0.00 (0.00, 0.00)		3.60 ± 5.70	6.92 ± 7.09	−3.32 (−10.22, 3.58), 0.319		7.85 ± 8.98	7.70 ± 8.88	0.15 (−9.42, 9.73), 0.97	
	mean change (95% CI) from baseline, *p* value					3.60 (−1.28, 8.47), 0.136	6.92 (2.04, 11.80), 0.009			7.85 (1.08, 14.63), 0.026	7.70 (0.93, 14.47), 0.029		
Secondary Outcomes	FMA-UE	16.38 ± 10.14	18.63 ± 9.74	−2.25 (−12.1, 8.41), 0.66		27.38 ± 9.77	28.00 ± 13.46	−0.63 (−13.24, 11.99), 0.92		31.63 ± 12.03	29.75 ± 14.22	1.875 (−12.25, 16.00), 0.78	
	mean change (95% CI) from baseline, *p* value					11.00 (5.10, 16.0), 0.001	9.38 (3.47, 15.28), 0.004			15.25 (7.64, 22.86), 0.001	11.13 (3.51, 18.74), 0.007		
	extensor carpi radialis muscle	0.00 ± 0.00	0.25 ± 0.46	−0.25 (−0.60, 0.10), 0.149		1.00 ± 0.76	1.25 ± 1.04	−0.25 (−1.22, 0.72), 0.590		1.50 ± 0.76	1.13 ± 0.83	0.38 (−0.48, 1.23), 0.362	
	mean change (95% CI) from baseline, *p* value					1.00 (0.30, 1.70), 0.009	1.00 (0.30, 1.70), 0.009			1.50 (0.90, 2.10), 0.000	0.88 (0.27, 1.48), 0.008		
	extensor carpi ulnaris muscle	0.00 ± 0.00	0.13 ± 0.35	−0.13 (−0.39, 0.14), 0.334		0.63 ± 0.52	0.25 ± 0.71	0.38 (−0.29, 1.04), 0.246		0.63 ± 0.52	0.50 ± 0.76	0.13 (−0.57, 0.82), 0.705	
	mean change (95% CI) from baseline, *p* value					0.63 (0.10–1.15), 0.023	0.13 (−0.40, 0.65), 0.619			0.63 (0.14, 1.11), 0.015	0.38 (−0.11, 0.86), 0.12		
	extensor digitorum communis muscle	0.00 ± 0.00	0.00 ± 0.00	0.00 (0.00, 0.00)		0.63 ± 0.92	0.38 ± 0.74	0.25 (−0.65, 1.15), 0.559		1.00 ± 0.93	0.50 ± 0.76	0.50 (−0.41, 1.41), 0.256	
	mean change (95% CI) from baseline, *p* value					0.63 (−0.01, 1.26), 0.053	0.38 (−0.26, 1.01), 0.224			1.00 (0.36, 1.64), 0.005	0.50 (−0.14, 1.14), 0.116		
	BI	49.38 ± 14.74	48.13 ± 21.37	1.25 (−18.44, 20.94), 0.894		64.38 ± 18.79	61.88 ± 18.89	2.50 (−17.70, 22.70), 0.795		75.63 ± 10.84	68.13 ± 13.08	7.50 (−5.38, 20.38), 0.232	
	mean change (95% CI) from baseline, *p* value					15.00 (5.10, 24.90), 0.006	13.75 (3.85, 23.65), 0.01			26.25 (17.80, 34.70), 0.000	20.00 (11.55, 28.45), 0.000		

EMGB: electromyography bridge; NMES: neuromuscular electrical stimulation; AROM: active range of motion; FMA-UE: Fugl-Meyer Assessment for Upper Extremity; BI: Barthel Index. Values are presented as the number of patients (*n*) or mean ± standard deviation.

## Data Availability

The data presented in this study are available on request from the corresponding authors.

## Supplementary Materials

The following supporting information can be downloaded at: https://www.mdpi.com/article/10.3390/brainsci12070870/s1, Table S1: Demographic and Clinical Information for Patients; Table S2: Demographic and clinical information for Patients (long course Removed); Table S3: Clinical Characteristics of All the Patients in Both Groups.
