# Supplementary material for: Effects of Electromyography Bridge on Upper Limb Motor Functions in Stroke Participants: An Exploratory Randomized Controlled Trial
Source: Brain Sci. 2022 Jun 30;12(7):870. doi: 10.3390/brainsci12070870 (PMC9312916; doi:10.3390/brainsci12070870)
Supplement: Supplementary file 1 [file brainsci-12-00870-s001.zip › brainsci-1771988-supplementary.pdf]

Supplementary Material

Table S1. Demographic and Clinical Information for Patients.

Patient No.	Sex/Age, year	Stroke Onset, month	Group	Hemiplegia side	Type of Stroke	HAMD
45	F/72	9	EMGB	L	hemorrhagic	4
47	F/70	1	EMGB	L	ischemic	0
51	F/73	4	EMGB	L	ischemic	0
54	M/43	1	EMGB	R	hemorrhagic	1
53	F/68	1	EMGB	L	ischemic	9
39	F/73	9	EMGB	L	hemorrhagic	1
56	F/51	6	EMGB	R	hemorrhagic	0
60	M/25	1	EMGB	L	hemorrhagic	10
46	M/55	1	EMGB	L	ischemic	0
59	M/51	1	EMGB	L	hemorrhagic	18
41	M/38	5	EMGB	L	hemorrhagic	0
38	M/35	5	EMGB	R	hemorrhagic	1
48	M/68	8	EMGB	L	hemorrhagic	2
55	M/37	1	EMGB	R	hemorrhagic	0
42	M/55	1	NMES	L	hemorrhagic	3
58	F/73	1	NMES	L	hemorrhagic	2
52	F/51	1	NMES	L	ischemic	18
43	M/52	1	NMES	R	hemorrhagic	10
44	F/35	4	NMES	R	hemorrhagic	0
50	M/60	1	NMES	L	ischemic	0
57	M/56	2	NMES	L	hemorrhagic	11
40	M/49	2	NMES	R	hemorrhagic	0

M: male; F: female; EMGB: electromyography bridge; NMES: neuromuscular electrical stimulation; HAMD: Hamilton Rating Scale for Depression.

Table S2. Demographic and clinical information for Patients (long course Removed).

Patient No.	Sex/Age, year	Stroke Onset, month	Group	Paralyzed Side	Type of Stroke	HAMD
47	F/70	1	EMGB	L	ischemic	0
51	F/73	4	EMGB	L	ischemic	0
54	M/43	1	EMGB	R	hemorrhagic	1
53	F/68	1	EMGB	L	ischemic	9
60	M/25	1	EMGB	L	hemorrhagic	10
46	M/55	1	EMGB	L	ischemic	0
59	M/51	1	EMGB	L	hemorrhagic	18
48	M/68	8	EMGB	L	hemorrhagic	2
55	M/37	1	EMGB	R	hemorrhagic	0
42	M/55	1	NMES	L	hemorrhagic	3
58	F/73	1	NMES	L	hemorrhagic	2
52	F/51	1	NMES	L	ischemic	18
43	M/52	1	NMES	R	hemorrhagic	10
44	F/35	4	NMES	R	hemorrhagic	0
50	M/60	1	NMES	L	ischemic	0
57	M/56	2	NMES	L	hemorrhagic	11
40	M/49	2	NMES	R	hemorrhagic	0

M: male; F: female; EMGB: electromyography bridge; NMES: neuromuscular electrical stimulation; HAMD: Hamilton Rating Scale for Depression.

Table S3 . Clinical Characteristics of All the Patients in Both Groups.

	EMGB group (n=14)	NMES group (n=8)	*P value*
**Age**	54.21±16.60	53.88±10.70	0.954
**Sex, *n* (%)**			
**Male**	8(57.1)	5(62.5)	1
**Female**	6(42.9)	3(37.5)	
**Stroke type, *n* (%)**			
**Infarction**	4(28.6)	2(0.25)	1
**Hemorrhage**	10(71.4)	6(0.75)	
**Paralyzed side**			
**Left**	10(71.4)	5(62.5)	1
**Right**	4(28.6)	3(37.5)	
**HAMD**	3.29±5.38	5.50±6.72	0.406
**Stroke onset, month**	3.79±3.21	1.63±1.06	0.034

Abbreviations: EMGB, electromyography bridge; NMES, neuromuscular electrical stimulation; HAMD, Hamilton Rating Scale for Depression.

*Values are presented as the number of patients (n) or mean±standard deviation.

Significance difference at $p < 0.05$.
